# Attentional Conflict Moderates the Association Between Anxiety and Emotional Eating Behavior: An ERP Study

**DOI:** 10.3389/fnhum.2018.00194

**Published:** 2018-05-15

**Authors:** Greg Denke, Eric Rawls, Connie Lamm

**Affiliations:** ^1^School of Social and Behavioral Sciences, Casper College, Casper, WY, United States; ^2^Department of Psychological Science, J. William Fulbright College of Arts & Sciences, University of Arkansas, Fayetteville, AR, United States

**Keywords:** emotional eating, N2 activation, attentional conflict, anxiety, attentional blink, RSVP task

## Abstract

Emotional eating is an attempt to avoid, control, or cope with negative emotions through eating a large amount of calorie dense sweet and/or high fat foods. Several factors, including various attentional mechanisms, negative affect, and stress, impact emotional eating behavior. For example, attentional narrowing on negative events may increase attentional stickiness and thereby prevent the processing of more peripheral events, such as eating behavior. This study contributes to the extant literature by examining the neural correlates underlying the attentional conflict between processing negative events and regulating behavior within a task that emulates how negative life experiences might contribute to unrestrained eating behavior. We explore this question within a normative sample that varies in their self-reported anxiety symptoms. Dense-array EEG was collected while participants played the attentional blink game—a task in which excessive attentional resource allocated to one event (e.g., negative picture) interferes with the adequate attentional processing of a second event that requires action. To assess the attentional conflict, we measured N2 activation, an event-related potentials (ERPs; averaged EEG) associated with conflict processing. Results revealed that N2 activation moderates the association between anxiety and emotional-eating behavior. Thus, increased anxiety combined with more negative N2 activation can contribute to emotional-eating behavior. These results are discussed in the context of ineffective conflict processing contributing to poor emotion regulation.

## Introduction

Emotional eating is an attempt to avoid, control, or cope with negative emotions through eating a large amount of calorie dense sweet and/or high fat foods (Macht et al., 2002; Konttinen et al., 2010; van Strien et al., 2013). Eating as a means to deal with stress and anxiety is a widespread, maladaptive, emotion regulation/coping behavior that is a momentary fix at best and often leads to additional problems. According to the American Psychological Association’s (2014) *Stress in America* survey, 38 percent of adults reported that they had engaged in emotional eating within the past month, with 49 percent of them doing it weekly. Though this behavior is a means to deal with negative emotions, rather than eliminating these problems, it typically results in additional stress. Emotional eaters often feel guilty about their eating behavior, feel sluggish, and/or feel bad about their physiques immediately after emotional eating episodes (American Psychiatric Association, 2013). Additionally, emotional eating can lead to a clinical level of dysfunction, i.e., binge-eating disorder (BED; Stice et al., 2002; Masheb and Grilo, 2006) and is a contributing factor for weight gain and obesity (Geliebter and Aversa, 2003; Croker et al., 2011).

A number of factors contribute to emotional eating problems (e.g., Nolen-Hoeksema et al., 2007; Kubiak et al., 2008; Kemp et al., 2011), including several attentional mechanisms (Nolen-Hoeksema et al., 2007; Kubiak et al., 2008; Kemp et al., 2011) as well as elevated negative affect and stress (Lingswiler et al., 1989; Grange et al., 2001). Negative affect, such as anxiety, has been implicated as a primary trigger for emotional-eating episodes (Waters et al., 2001), often interfering with cognitive control over eating behavior (Engelberg et al., 2007). Additionally, Heatherton and Baumeister (1991) suggest that a narrowing of attention disengages normal inhibitions against eating by focusing on an exclusive small range of stimuli. It may be that emotional-eaters narrow their attention on current negative events and that this attentional stickiness prevents thoughts about ongoing dietary restraints and thus contributes to emotional-eating episodes. This theory is supported by the fact that several studies have found a strong link between emotional-eating behavior and attentional rumination (Fairburn et al., 2003; Nolen-Hoeksema et al., 2007; Kubiak et al., 2008). Thus, negative affect, such as anxiety, might lead to attentional stickiness/rumination on negative events, thereby, inducing attentional conflict between the negative events and subsequent moment-to-moment routine response requirements, such as inhibiting eating unhealthy food.

The current study explores if attentional conflict between task demands and previous negative events contributes to emotional eating behavior using event-related potentials (ERPs). Specifically, we examined if N2 activation, a mediofrontal ERP measured 200–400 ms after a stimulus that requires conflict processing and that has been associated with response conflict and conflict monitoring (van Veen and Carter, 2002; Nieuwenhuis et al., 2003; Donkers and van Boxtel, 2004; Bartholow et al., 2005; Dimoska et al., 2006). Additionally, the N2 has been found to be sensitive to the emotional context of events (Lewis et al., 2006; Lamm et al., 2012, 2013). For example, Lewis et al. (2006) found emotion-related changes in N2 activation within a go/no-go task designed to induce negative emotion. Additionally, Lamm et al. (2012) found greater N2 activation in response to emotionally salient stimuli in comparison to neutral stimuli in the context of a go/no-go task. Greater N2 activation has also been associated with elevated trait anxiety (Sehlmeyer et al., 2010). Thus, it may be that anxious individuals who get attentionally stuck to negative events show greater (more negative) N2 activation, likely due to increased attentional conflict, and thus show elevated emotional eating behavior. We predicted that N2 activation would moderate the anxiety-emotional eating behavior relationship. More specifically, that anxious individuals with more negative N2 activation would show the greatest amount of emotional eating behavior but that anxious individuals who showed less negative N2 activation would not show elevated emotional eating behavior.

To emulate a real-world environment of interacting with multiple events, we used a rapid serial visual presentation (RSVP) task that presented 17 negative and neutral pictures in close succession. It has been found that when an individual focuses a large amount of their attentional resources on a particular item (e.g., an emotionally charged image), there is an increased likelihood that a subsequent item will not be recognized (Raymond et al., 1992; Most et al., 2005). This phenomenon, referred to as an attentional blink (AB), is believed to be the result of using a large amount of attentional resources for the processing of one stimulus, resulting in suboptimal processing of subsequent stimuli. An AB can be reliably measured using an RSVP task in which two stimuli are imbedded and separated by roughly 200–500 ms (Raymond et al., 1992; Vogel et al., 1998; Di Lollo et al., 2005; Shapiro et al., 2006).

## Materials and Methods

### Participants

Participants were undergraduate students (normative sample; *N* = 114; 76 female, 38 male; mean age = 22.50, *SD* = 5.49; right handed = 99, left handed = 15) who attended the University of New Orleans. All participants had normal or corrected-to-normal vision and were free of current psychiatric diagnoses. One participant was excluded from the analysis due to missing questionnaire data. Fifty-one participants who did not have a sufficient amount of trials to make an ERP, due to artifacts or poor performance, were also excluded from the analysis. Excluded participants did not significantly differ from included participants in age *t*(111) = 0.12, *p* = 0.96, or sex *t*(112) = 0.79, *p* = 0.43. Participants were recruited through undergraduate classes and earned course credit for their participation. This study received IRB approval from the University of New Orleans Institutional Review Board. Participants have previously been described in the first author’s master’s thesis (Denke, 2014).

### Measures

Measures have previously been described in the first author’s master’s thesis (Denke, 2014).

#### Dutch Eating Behavior Questionnaire (DEBQ)

The Dutch Eating Behavior Questionnaire (DEBQ) is a measure of eating behavior (van Strien et al., 1986). The DEBQ consists of three subscales: emotional eating, restraint, and externality scales, for a total of 33 items answered on a 5-point Likert scale ranging from “never” to “very often”(van Strien et al., 1986). Only the 13 items from the emotional eating subscale were used in the present investigation.

#### Emotional Eating Scale (EES)

The Emotional Eating Scale (EES) is a measure of emotion induced eating behavior (Arnow et al., 1995). The EES consists of 25 mood descriptions (e.g., bored or nervous) answered on a 5-point Likert scale ranging from “no desire to eat” to “an overwhelming urge to eat” (Arnow et al., 1995). After reading the mood description (e.g., irritated), participants indicate what their usual desire to eat level would be (e.g., an overwhelming urge to eat).

#### Binge-Eating Behavior

A binge-eating behavior composite score was generated for each participant by averaging the standardized scores for the DEBQ and EES measures. These two measures were found to be strongly correlated, *r*(114) = 0.43, *p* < 0.001.

#### State-Trait Anxiety Inventory Form Y (STAI)

The State-Trait Anxiety Inventory Form Y (STAI) is a measure of both trait and state anxiety in adults (Spielberger et al., 1983). The STAI consists of 20 items for assessing trait anxiety and 20 items for assessing state anxiety. The items ask questions about usual affect (Trait) or current affect (State) and are answered on a 4-point scale ranging from “Almost Never” to “Almost Always.”

#### Attentional Blink Task

Raymond et al. (1992) introduced the term AB, a psychological construct in which attention is momentarily inaccessible due to the processing of previous information. When two targets are to be identified among non-target distractors most individuals show an AB in reporting the second target. Correct identification of the first target (T1) impedes the detection of a second target (T2) that appears within 500 ms of T1 (Raymond et al., 1992; Chun and Potter, 1995). The failure to report a T2 is believed to happen because a large amount of attentional resources have been allocated to T1 (Shapiro et al., 2006). The AB is induced when salient stimuli cause a focus of attention (Shapiro et al., 2006). Moreover, Olivers and Nieuwenhuis (2005) found that the size of an AB is determined by an individual’s psychological state and that a strong focus on T1 promotes the AB. Shapiro et al. (2006) found that performance on T2 could be predicted from the amount of resources used in the processing of T1; more resources used for T1 equated to larger blink magnitude for T2.

**FIGURE 1:**
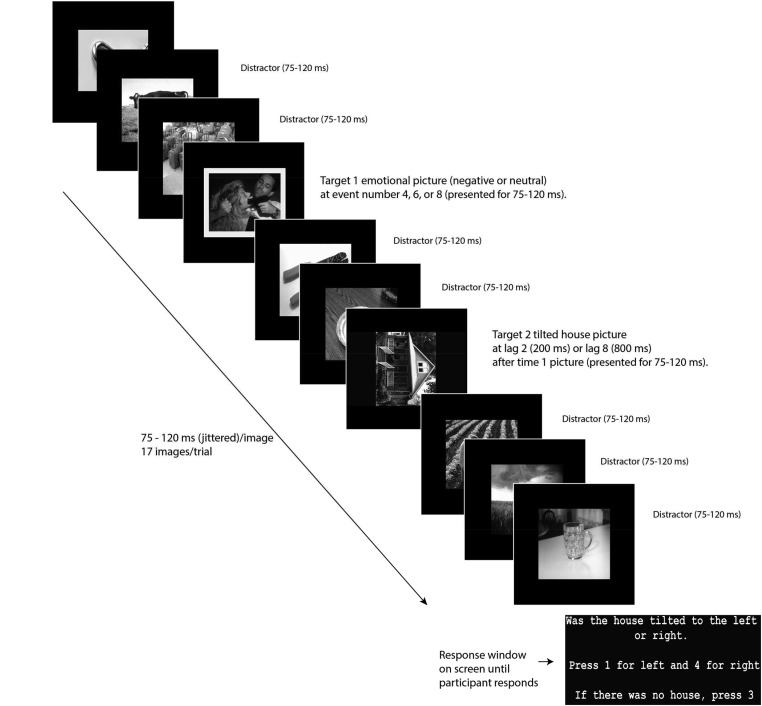
Task diagram. Reprinted from Denke (2014).

In line with Denke (2014), in the current task (**Figure 1**), participants began with a 10-trial practice session with instructions emphasizing that T2 will follow the picture with the yellow frame (T1). The task consisted of four blocks of 120 trials each. At the end of each trial, participants pressed either “1” for a house tilted left, “4” for a house tilted to the right or “3” if no house was seen. To prevent participants from looking at their hands to indicate the correct button, which would lead to EEG eye artifact, button 3 was marked by a large fuzzy sticker that could easily be identified by touch alone.

Between each block of the task, participants were instructed to stretch and blink their eyes in order to get comfortable and ready to proceed with the next block. Stimuli were black and white photographs: 120 T1 images framed in yellow (60 negative T1 images and 60 neutral T1 images), 200 neutral distractor images (each used randomly 9 times), and 100 T2 images (house photos, 50 tilted 90° to the left and 50 tilted 90° to the right) all presented on a 34 cm wide × 27 cm high LCD monitor. We also included 20 trials where no T2 image was presented to make missed T2 trials a true option and thereby prevent random responding when T2 was not observed. Emotional and neutral pictures were drawn from the International Affective Picture System (IAPS; Lang et al., 2008). House pictures were drawn from publicly available sources. Negative IAPS pictures were of people or animals and included graphic images of violence and mutilation. The neutral pictures were balanced with the negative pictures for numbers of depictions of people and animals. Trials consisted of a RSVP stream of 17 images, presented for 75–120 ms, and jittered trial-by-trial to aid in ERP processing. Depending on the trial, T1 was presented as the 4th, 6th, or 8th stimulus. T2 was presented either two or eight pictures after the T1 (lag 2 and lag 8). Preliminary analysis showed significantly greater performance accuracy for Lag 8 trials than Lag 2 trials, *t*(113) = 6.97, *p* < 0.001 (negative), *t*(113) = 5.48, *p* < 0.001 (neutral), indicating that our task showed the canonical AB phenomenon.

### Procedure

Upon arrival, participants were given a tour of the laboratory. Once all their questions were answered, written consent was obtained. Next, questionnaires were completed and then the electrode sensor net was applied to the participants’ heads. The participant’s chair was arranged so that they were seated 67 cm from a computer monitor. Task instructions were given and participants completed a practice block, identical to the main task, of 10 trials. When proficiency was shown on the practice block participants went on to perform the actual task. On average, the task took 30 min to complete.

### EEG Data Collection and Analysis

**FIGURE 2:**
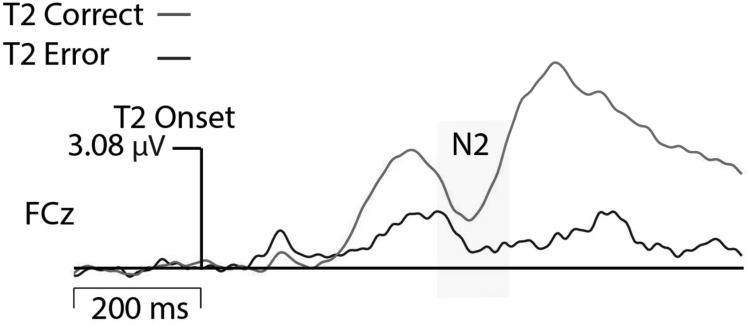
ERP waveform time-locked to T2 house presentation (neutral condition).

EEG was recorded using a 128-channel Geodesic Sensor Net and sampled at 250 Hz, using EGI software (Net Station; Electrical Geodesics, Inc., Eugene, OR, United States). Data acquisition was started after all impedances for all EEG channels were reduced to below 50 kΩ. All channels were referenced to Cz (channel 129) during recording and were later re-referenced against an average reference corrected for the polar average reference effect (PARE correction; Junghöfer et al., 1999). Data was filtered using a FIR bandpass filter with a low-pass frequency of 50 Hz and a high-pass frequency of 0.3 Hz. To best capture eye blink artifacts, the threshold was set to 140 μV threshold (peak-to-peak) and all trials in which this threshold was violated were excluded from analyses. Furthermore, signal activation change (peak-to-peak) exceeding 100 μV across the entire segment were marked as bad and interpolated. N2 amplitude data were time-locked to the T2 and baseline corrected to 200 ms before T2 onset. Data from trials with correct T2 detection and trials in which T2 was erroneously not detected were analyzed for Lag 2 trials. Grand averaged data showed that N2 activation was greatest at electrode FCz; therefore, activation for FCz and surrounding midline electrodes were exported. To allow for individual differences in peak activation, the most negative N2 activation across this small cluster of mediofrontal electrodes was analyzed. N2 activation was greatest between 340 and 470 ms after T2 stimulus onset (see **Figure 2**); therefore, peak activation was exported for this time range. The average number of trials comprising ERPs in the neutral correct condition was 57 (range: 21–84), neutral error 28 (range: 10–60), negative correct 61 (range: 23–84), and negative error 23 (range: 10–58). All N2 and behavioral data that showed values greater or less than 2 SD from the mean were modified to reflect exactly 2 SDs from the mean (outlier correction) thereby preventing statistical analyses from being skewed by outliers.

### Data Analyses

Since increased anxiety does not always lead to increased emotional eating behavior (our measures only had a correlation, *r*(63) = 0.21, *p* = 0.1), regression analyses were conducted to examine the moderating role of conflict related brain activation on the relation between anxiety and emotional-eating behavior. *A priori t*-tests revealed sex differences for some independent and dependent variables (**Table 1**); therefore, effects of sex were controlled for by entering sex in the first step of all N2 regression analyses. Additionally, for data in the negative error condition, negative correct brain data were entered in the first regression step to control for unrelated neural activation. For data in the neutral error condition, neutral correct brain data were entered in the first regression step to control for unrelated neural activation. Data for all conditions are comprised of N2 amplitudes (we did not analyze any difference waves or difference scores).

**Table 1:** Sex differences.

Measures	Female mean	Male mean	*t*(61)	*p*
				
Negative correct N2	-0.82	-0.11	1.68	0.10
Neutral correct N2	-1.56	-0.53	2.19	0.03
Negative error N2	-2.84	-1.73	2.05	0.05
Neutral error N2	-2.85	-2.14	1.54	0.13
Emotional Eating Scale	1.96	2.14	1.10	0.28
Dutch Eating Behavior	2.49	2.51	0.15	0.88
EES-DEBQ Composite	-0.13	0.05	0.74	0.46
STAI trait	43.90	43	-0.37	0.71
STAI state	34.38	33	-0.64	0.53

## Results

### Behavioral Analysis of the Attentional Blink Phenomenon

**FIGURE 3:**
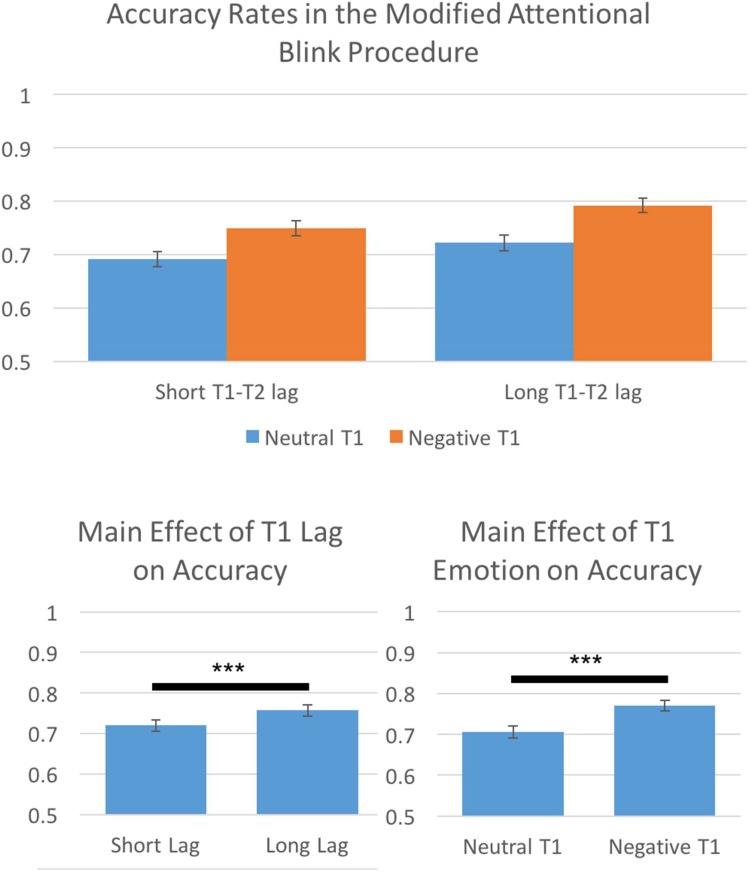
Behavioral results indicated that the prototypical Attentional Blink effect was present, participants were more accurate for long T1 to T2 lags than for short T1 to T2 lags. Furthermore, participants were more accurate for negative T1 trials than neutral T1 trials. ^∗∗∗^*p* < 0.001.

The AB has been shown to be greatest when T2 falls shortly after T1 (roughly 200–500 ms) and that later time intervals show much lower levels of ABs (higher performance accuracy; e.g., Martens and Wyble, 2010). We conducted a 2(Lag Time: short T1 to T2 interval, long T1 to T2 interval) by 2(T1 Emotion: neutral, negative) repeated-measures ANOVA on performance accuracy to assess if we effectively captured the AB. Results indicated significant main effects of both Lag Time, *F*(1,113) = 71.31, *p* < 0.001, η^2^ = 0.39, 𝜀 = 1.0, and T1 Emotion, *F*(1,113) = 171.18, *p* < 0.001, η^2^ = 0.60, 𝜀 = 1.0. Consistent with the AB literature, we found that long T1 to T2 intervals were more accurate than short T1 to T2 intervals. Additionally, participants showed higher accuracy for negative T1 trials than neutral T1 trials (**Figure 3**).

### ERP Moderator Effects

Linear regression analyses were conducted to determine whether neural activation during an AB (i.e., the commission of an error by responding that there was no house when a house was present) moderated the association between anxiety scores (STAI) and emotional eating behavior (EES & DEBQ Composite score). For all regression analyses, sex and baseline activation (N2 activation for correct trials in the same emotional condition; as mentioned above in section “Data Analyses”) were entered in the first step; independent variable (STAI trait or STAI state) and N2 amplitude in the error condition were entered on the second step; and an interaction term of STAI and N2 amplitude (error condition; standardized to decrease the possibility of multicollinearity or scaling influencing results, Dawson, 2014) was entered in step 3 to test for moderation effects. Emotional eating composite score was entered as the dependent variable.

**FIGURE 4:**
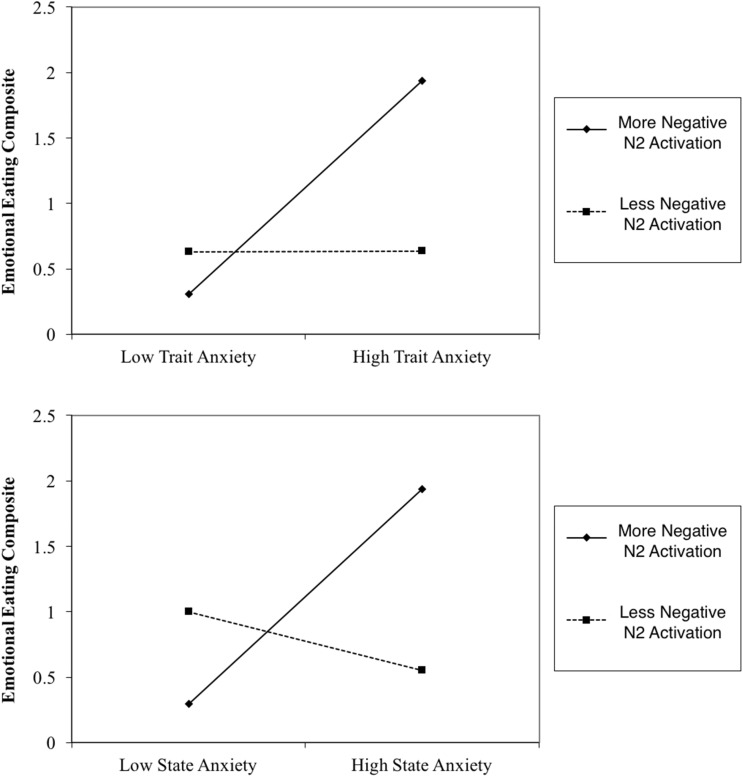
N2 Moderation Plots. More negative N2 activation refers to a more negative deflection in N2 amplitude.

Results indicated that N2 activation in neutral contexts significantly moderated the association between (1) STAI trait and emotional eating behavior, β = -0.41, *t*(56) = -2.69, *p* = 0.009 and (2) STAI state and emotional eating behavior, β = -0.52, *t*(56) = -3.08, *p* = 0.003. When probed at values of 1 SD above and below the mean, additional regression analyses revealed that at high levels of N2 activation (more negative), anxiety was a significant predictor of emotional eating scores, Trait: β = 0.82, *t*(56) = 3.30, *p* = 0.002, State: β = 0.82, *t*(56) = 3.62, *p* = 0.001. However, at low levels of N2 activation (less negative), anxiety was not a significant predictor of emotional eating scores, Trait: β = 0.002, *t*(56) = 0.02, *p* = 0.99, State: β = -0.22, *t*(56) = -1.25, *p* = 0.22. N2 moderation plots are presented in **Figure 4**. Critically, this moderating effect cannot be attributed to overall attentional or inhibitory control abilities, overall negative affect, or age, as follow-up analyses with these factors added in the first step of the regression model revealed that none of these factors altered the pattern of significance present. Therefore, it is likely that our moderating effect is indeed an interaction between anxiety and underlying neural activation, and cannot be attributed to various global factors.

Furthermore, counter to our hypothesis, N2 activation in negative contexts did not moderate the association between either STAI trait or STAI state and emotional eating scores.

## Discussion

The current study examined if conflict processing moderated the association between anxiety and emotional eating behavior. We measured N2 activation, an ERP associated with conflict processing (van Veen and Carter, 2002; Nieuwenhuis et al., 2003; Donkers and van Boxtel, 2004; Bartholow et al., 2005; Dimoska et al., 2006), in the context of an AB task. More specifically, we explored if individuals high in anxiety symptoms required larger amounts of conflict-related neural activation (N2 activation) in order to respond to events that occur after an attention-grabbing event.

Results revealed that individuals revealing high levels of anxiety symptomatology who also recruited large amounts of N2 activation (more negative) showed high levels of emotional eating behavior. To the best of our knowledge, no previous research has addressed this question. These results are in line with much of the N2 anxiety literature (e.g., Righi et al., 2009; Sehlmeyer et al., 2010; Hum et al., 2013), which consistently shows more negative N2 activation for individuals with elevated anxiety. For example, Sehlmeyer et al. (2010) showed this association for individuals with high levels (compared with low levels) of trait anxiety in the context of a go/no-go task and Hum et al. (2013) found the same pattern of effects for children with clinical levels (compared to non-clinical controls) of generalized anxiety disorder, social anxiety disorder, or separation anxiety disorder also in the context of a go/no-go task. Additionally, consistent with our results, these effects are frequently found in the context of unemotional stimuli (e.g., Mohlman et al., 2007; Yoon and Zinbarg, 2008; Hum et al., 2013). Together, this pattern of elevated neural activation in seemingly neutral contexts has been interpreted to reflect an indiscriminate, over-generalized and excessive effort to regulate (Hum et al., 2013; Lamm et al., 2014).

Combining the previous discussion with Heatherton and Baumeister’s (1991) theory that attentional narrowing contributes to emotional eating behavior, it may be that anxious individuals may get attentionally “stuck” on key events and therefore experience heightened attentional conflict between this event and subsequent events. We hypothesized that this effect would be greatest in the context of negative images because emotional eating behavior has consistently been associated with attentional rumination (e.g., Fairburn et al., 2003; Nolen-Hoeksema et al., 2007; Kubiak et al., 2008). Counter to our hypothesis, we did not find this moderating effect for negative trials. It may be that in the context of negative violent images all participants showed greater N2 activation and therefore we may not have had enough variance to reveal moderation. This hypothesis is supported by the fact that numerous studies have shown increased N2 activation in the context of negative images irrespective of individual differences or disorders (e.g., Li et al., 2008; Lamm et al., 2012). Given that anxious people seem to exhibit heightened regulation even in unemotional contexts (Hum et al., 2013; Lamm et al., 2014) and the potential restriction of variance in the context of negative emotion (outlined above), together, these arguments might explain why we only found that the N2 moderated the anxiety – emotional eating relationship in relatively neutral contexts. Given that N2 activation specifically (Lewis et al., 2008) and cognitive control more generally (e.g., Braver et al., 2009) has been shown to change with practice, it may be that with conflict monitoring/resolution practice, emotional eaters can learn to regulate more efficiently and thereby prevent lapses in regulation leading to emotional-eating behavior.

### Limitations

The current study has several limitations. First, it is important to note, that in this current study, we use a laboratory environment to emulate real world negative stimuli induced ABs. While this is a useful approach to exploring the neural correlates underlying attentional conflict and how it may contribute to emotional eating behavior, these pictures are not as salient as real-world emotions. Thus, the current project has a much smaller temporal scale than might be found for real world emotion-induced eating.

Second, within the AB literature, there are two different types of AB tasks: (1) the task requires only a single response to the second target event (e.g., Most et al., 2005) and (2) the task requires two responses, one to the first target and one to the second target (e.g., Shapiro et al., 2006). To the best of our knowledge, there is no meta-analysis outlining which approach is better. Therefore, we chose the type of task that had previously been used for emotion-induced ABs (Most et al., 2005). Future research should explore the association between anxiety, conflict processing, and emotional eating using a dual response task.

## Conclusion

The current study shows that individuals who show higher levels of anxiety symptoms and who ineffectively apply conflict processing show heightened emotional-eating behavior. These are novel findings that build on the anxiety literature and highlight the need to address the ability to process conflict when treating emotional eating behavior and obesity. Future research should replicate these findings in a clinical binge eating disorder sample.

## Ethics Statement

This study was carried out in accordance with the recommendations of the Institutional Review Board at the University of New Orleans with written informed consent from all subjects. All subjects gave written informed consent in accordance with the Declaration of Helsinki. The protocol was approved by the Institutional Review Board at the University of New Orleans (protocol: IRB06Dec12).

## Author Contributions

CL is the PI of the project and oversaw the design and execution of the project. She also wrote part of the introduction and discussion. GD designed the study and collected most of the data and also wrote parts of the introduction, materials and methods, and discussion. ER analyzed the data and wrote the results section as well as part of the materials and methods section.

## Conflict of Interest Statement

The authors declare that the research was conducted in the absence of any commercial or financial relationships that could be construed as a potential conflict of interest.
